# Supplementary material for: Patterns of Intron Gain and Loss in Fungi
Source: PLoS Biol. 2004 Nov 30;2(12):e422. doi: 10.1371/journal.pbio.0020422 (PMC532390; doi:10.1371/journal.pbio.0020422)
Supplement: Table S1 — Also available at http://genes.mit.edu/NielsenEtAl/. (4.3 MB ZIP). [file pbio.0020422.st001.zip › NielsenEtAl/html/1410.html]

AN1969.1.NCU06762.1.MG07163.1.FG04412.1


```
 CLUSTAL W (1.82) Multiple Sequence Alignments - Introns Inserted


Sequence 1: MG07163.1	331 aa
Sequence 2: FG04412.1	316 aa
Sequence 3: NCU06762.1	324 aa
Sequence 4: AN1969.1	302 aa
Alignment Length: 349 aa
Number Identitical Residues: 87 aa
Alignment Score (without introns) 5571


MG07163.1 	MHLPYPARKDSNAARYKPARPPSASSGYIPPILRRIRLRGGLIIAVVTFACFYLFFSISR
NCU06762.1	MHFAYPPRKSSNPP------PFRPRTSALP-TIRGKRLKTIVLIGLAFLTLLWLFQRG--
FG04412.1 	MQFAYPPRKNSNSP------PFGRRSGKIPPILRRLRKRTILLGFMGIAGLLYLLFGS--
AN1969.1  	MQFAVPPRKGFSPA------PYARTS--FLTFQRRKQLKTIIILSVTFLAVFFLLSHP--
          	*::. *.**. ...      *    :  : .  *  : :  ::  :     ::*:     

MG07163.1 	ASTASRSIKRSGAQHPITTHDPTGNPPAVLVTVMDSGLHSNSYLQNLKENRLTYAKKH-~
NCU06762.1	---------GRSRHAPPAYHKPSGNPPVVIVTVFNEGKYGKGYLDMVKENRLKYAEKHA1
FG04412.1 	---------KSGS--PYHEHVPSGNPPVVVVTVSDYSEYSTSYLESIRNNREQYAKRH-~
AN1969.1  	-----------FYPSIRTAAGPVGSPGVVIVTLLDRAMYSDTYLQKIIKNREDYAQRH-~
          	                     * *.* .*:**: : . :.  **: : :**  **::*  

MG07163.1 	---------G1YETWFPDVFEYDLK~GAPTSWSKVVAMRHALTKFPDATYFWFLDHDTII
NCU06762.1	YLPSSPPDPG~YGTFFVKSSDYDLR~GAPVSWATLPAVRHAMTEFPDASYIWYLDQNAFI
FG04412.1 	---------G1YEAMVVKVSDYNTG~ESPKSWARIMAMRHALSKYPDATYIWFLEQNAFI
AN1969.1  	----------~-------------G1NYPRSWAIVPAVRHAMASHPSATYFFHLDVHALF
          	                            * **: : *:***::..*.*:*::.*: .:::

MG07163.1 	VNMEPTVEQSLMEAKAIGDVMIKDHPVVPPDSIIKTFTHLKGEDVDLVLTQDKAGLSVGS
NCU06762.1	MNPQLKIEEHVMKPSRLEELMIKDHPVVPPDSIIKTFSHLKGHDVDFVLTQDKDGLSSSS
FG04412.1 	MELDKTLEEHVMAPATLESLMIRDWSVVPPDSIIKTFSHLKGQDVNLVISQDEVGLVTNS
AN1969.1  	MNSNESLEARLLNRHRLESLMRRDVPVVPPDSIIRTFSHLQPEDIDLIITSDAEDLSTGS
          	:: : .:*  ::    : .:* :* .********:**:**: .*::::::.*  .*  .*

MG07163.1 	FIVRNGEWAKFFLETWFDPIYRSYNFQKAEEHAL0EHIVQWHPTILSKLAIVPQRYINSY
NCU06762.1	FVVKNGEWAKFFLETWFDPIYRTYNFQKAEQHAL0EHIVQWHPTILAKLALVPQRILNSY
FG04412.1 	YVLRNGDWAKFFIETWMDPLYQSYNFEKAERHAL0EHIVQWHPTILSKLALIPQRTIASY
AN1969.1  	FVLKQGDFARFFLDTWFDPLYRSYNFAKAETHSL0EHIVQWHPTVLARMALVPQRVMNSY
          	:::::*::*:**::**:**:*::*** *** *:* *********:*:::*::*** : **

MG07163.1 	NTGDS----GESYQAGDMVVRFPACSKVGYPACEAEAAKYEKQWR---GTKS-
NCU06762.1	SKEKG----GQEYKEGDLAVRLYACGEAGEDTCETESQRFA-QWKAAYQDS--
FG04412.1 	GRNKE----GNAFREGDFVALMVDCTVTGQASCDKLSHIYQQQLRKKFGNEVA
AN1969.1  	SKESTRAAVDGTYKDGDSIIRFFGCDTDPNRDCEREMKPYYNLWATKVEIK--
          	.  .  :: .  :: **    :  *       *:     : .        .
```
